# Developing an integrated framework of healthcare leaders’ resilience

**DOI:** 10.1007/s11846-022-00572-2

**Published:** 2022-08-01

**Authors:** Charlotte Förster, Stephanie Duchek, Silke Geithner, Maxie Krägler

**Affiliations:** 1 Faculty of Economics and Business Administration, Junior Professorship of European Management, TU Chemnitz, 09111 Chemnitz, Germany; 2 Center for Responsible Research and Innovation, Fraunhofer Institute for Industrial Engineering IAO, 10623 Berlin, Germany; 3 Chair of Leadership and Organization Studies in the Social and Health Economy, University of Applied Sciences for Social Work, Education and Nursing Dresden, 01191 Dresden, Germany; 4 Faculty of Business and Economics, TU Dresden, 01062 Dresden, Germany

**Keywords:** Healthcare leaders, Healthcare leaders’ resilience, Healthcare leaders’ challenges, Workplace resilience, M12

## Abstract

Healthcare institutions have been under pressure for years now, climaxing in the COVID-19 crisis. Even if they are not operating at a trouble spot of the current COVID-19 pandemic, healthcare leaders need to be highly resilient to remain effective as well as staying healthy themselves. The purpose of this study is to shed light on healthcare leaders’ challenges and, more important, their specific resilience factors. By doing so, we can expand the knowledge of this fragmented research field and deliver an integrated framework that has so far been lacking. We conducted 20 in-depth interviews with healthcare leaders. More precisely, 35% (7) of our interviewees were physicians, 55% (11) worked in nursing, and 10% (2) were nonclinical professionals, with 50% (10) of our participants working in upper, 40% (8) in middle, and 10% (2) in lower leadership positions. Based on a qualitative content analysis approach, we identified main healthcare leaders’ challenges as well as crucial resilience factors (i.e., individual, situational, and behavioral factors). By integrating insights from contemporary leadership and work-related resilience research, we were able to develop an integrated framework of healthcare leaders’ resilience. Considering resilience as a context-dependent construct, we are contributing to the resilience and healthcare literature by investigating the specialty of healthcare leaders’ resilience. This study is contributing to the future development of resilience interventions in healthcare organizations that might help not only healthcare leaders to better cope with critical situations but also promote resilience development among their followers and organizations.

## Introduction

By producing rapidly rising numbers of new infections, deaths, overworking physicians and nurses, who report having to decide which patient lives or dies, the current COVID-19 crisis is challenging not only our healthcare systems but especially our healthcare workers. Insights from the SARS outbreak in 2003 suggest that the long-term psychological effects of this later crisis might be huge (e.g., Maunder et al. 2006; Tam et al. 2004), thus increasing the pressure on our already tense healthcare institutions. It is likely that the current pandemic will have a significant impact on healthcare workers in terms of burnout, emotional well-being, and moral distress (e.g., Hlubocky et al. 2021). Even today, nurses and physicians rank in the top of all occupations for having a high need of resilience (Dyrbye et al. 2019; Kossek and Perrigino 2016; West et al. 2020). Resilience, defined as *“*positive adaptation in the face of risk or adversity*”* (Wright et al. 2013: 17; Foerster and Duchek 2017, 2018), has been linked to better states of well-being beyond the simple absence of psychological disorders (e.g., Davydov et al. 2010; Foerster and Duchek 2017). Although resilience is certainly important for all healthcare professionals, it is particularly important for healthcare leaders since it is crucial not only for their own health and psychological well-being but also for the health and efficiency of their workforce (e.g., Foerster and Duchek 2017).

Despite the significance of this topic, research on healthcare leaders’ resilience seems to be widely neglected, especially in terms of a systematic overview of those factors that are crucial to resilience among healthcare leaders. To narrow this research gap, we conducted 20 in-depth, semi-structured interviews with healthcare leaders operating in different areas (e.g., medical services, nursing, nonclinical) and in different leadership positions (i.e., upper, middle, and lower leadership). All interviewees were leaders working in medical facilities situated in Germany. Based on resilience research in the leadership context (e.g., Foerster and Duchek 2017, 2018) and the work context (e.g., Cooper et al. 2013; Kossek and Perrigino 2016), our empirical study aimed to shed light on these questions: *What challenges do healthcare leaders perceive?* and *Which context-specific factors influence healthcare leaders’ resilience?* By relying on Foerster and Duchek’s (2017) systematization of leaders’ resilience factors and considering resilience as a context-dependent construct (e.g., Fletcher and Sarkar 2013; Kossek and Perrigino 2016), we developed an integrated framework of healthcare leaders’ resilience. By doing so, we are contributing to the resilience literature as well as to the healthcare literature by providing a comprehensive overview of relevant resilience factors (i.e., individual, situational, and behavioral factors) in this specific context. We are also contributing to the development of interventions that help leaders increase their resilience. Such interventions can enable healthcare leaders to cope effectively with minor everyday crises but also with crises that are more substantial. In addition, by promoting their own resilience, healthcare leaders not only improve their own well-being but also foster resilience development among their followers and organizations. Thus, our research contributes to efforts to create a resilient health care organization, a topic that has become increasingly important in light of the COVID-19 pandemic (Shanafelt et al. 2020). More specifically, our research also supports the “Quadruple Aim” of healthcare (Sikka et al. 2015), namely by “elevating clinician well-being to an integral institutional mission coupled with the triple aim to prioritize patient outcomes, costs, and experience” (Hlubocky et al. 2021: 368). The article starts with a clarification of what we already know about healthcare leaders’ resilience. After explaining the common ground as well as the method of this study, we present our findings and develop our integrated framework of healthcare leaders’ resilience. Following the discussion of these findings, we derive important practical implications.

## Theory

Considering the definition of resilience in the healthcare context, most articles refer to definitions already available in the prior literature. Resilience is described as an *ability* (e.g., Tau et al. 2018; Westcott 2016), a *capacity* (e.g., Smith and Wolf 2018; Van Gorder et al. 2015), a *quality* (e.g., Hudgins 2016; Tau et al. 2018), a *trait* (e.g., Buell 2014; Carpio et al. 2018), or a *process* (e.g., Carpio et al. 2018; Cline 2015). However, most authors refer to a general definition, with healthcare leaders’ resilience being seen as an ability to deal with adverse conditions that arise during their work (e.g., Tau et al. 2018).

Referring to healthcare leaders’ resilience, it is important to mention that it is a context-dependent construct (Fletcher and Sarkar 2013; Foerster and Duchek 2017), which means that resilience factors important to one group (e.g., children, employees, leaders) are not necessarily important to another group as well (e.g., healthcare leaders). The idea of resilience as a context-dependent construct (see also Ungar 2008) shows some overlaps with the approaches of contingency theory that emphasize the context or situation as decisive for different actions and strategies. Very well researched and confirmed is the context dependency in leadership research, according to which effects of leadership styles largely depend on the situation (e.g., Fiedler 1993; Yukl 2002; Yun et al. 2005). Considering the context dependency of resilience, some authors (e.g., Cooper et al. 2013; Kossek and Perrigino 2016) have depicted resilience in the workplace as the interaction of various individual and situational factors, thus recognizing the social environment in which the individuals interact. In particular, Kossek and Perrigino’s (2016) integrated occupational resilience framework outlines the meaning of occupational factors, whereby resilience can be both generalized across occupations as well as job specific. This will result in resilience forming a multilevel framework, as it is described as the “synthesis of an individual’s traits, capacities or coping strategies, and processes” that are used to positively adapt to adversity and risks originating from one’s organizational and occupational context (p. 764). Based on these insights, resilience can be understood as a process that depends on both individual and environmental features, which together contribute to what we understand as resilience outcomes, for instance, in terms of psychological well-being. Since we know that resilience is a context-dependent construct (e.g., Fletcher and Sarkar 2013), whereby the occupational and organizational contexts matter deeply (Kossek and Perrigino 2016; Hillmann 2021), findings cannot be simply transferred from one context to another.

Taking a closer look at articles examining resilience in health care, it is striking that although the work in the healthcare system spans several areas, most articles focus on nursing, with even fewer articles focusing on healthcare leaders. In more detail, the few papers that discuss healthcare leaders’ resilience focus on how resilience can be influenced and promoted. For this purpose, concepts are presented (Hudgins 2016; Kim and Windsor 2015), single factors that influence resilience are highlighted (Bernard 2019; Kelly et al. 2016; Koen et al. 2013; OʼConnor and Batcheller 2015), and concrete guides, protocols, or practices related to resilience development are introduced (Bright 1997; Buell 2014; Hamilton 2015; Smith and Wolf 2018; Wicks and Buck 2013). To give an overview of the factors potentially influencing healthcare leaders’ resilience, we extracted them from the previous healthcare leaders’ resilience literature, and subdivided them into internal, external, and organizational factors (see Table 1).

**Table 1:** Factors of healthcare leaders’ resilience

	Source
*Internal* factors of healthcare leaders’ resilience	
(physical) health (= fundamental)	Buell (2014), Koen et al. (2013)
hope and optimism (= beneficial attitudes)	Bright (1997), Buell (2014), Koen et al. (2013), Wicks and Buck (2013)
positive thinking	Bernard (2019)
openness, willingness to learn and realism	Buell (2014), Smith and Wolf (2018)
positive emotions (joy, interest, satisfaction, pride, love) (= broaden-and-build theory)	Tugade and Fredrickson (2004), Hamilton (2015)
self-awareness, self-efficacy and self-care (also described as self-respect, self-esteem and self-knowledge)	Bernard (2019), Bright (1997), Buell (2014), Hudgins (2016), OʼConnor and Batcheller (2015), Smith and Wolf (2018)
professional knowledge (medical and in business)	Kelly et al. (2016)
communication and problem-solving skills	Koen et al. (2013), Smith and Wolf (2018)
emotional intelligence (EI)	Cline (2015), Hamilton (2015), Hudgins (2016), Akerjordet and Severinsson (2008)
reflection	Cline (2015), Hamilton (2015)
seeking feedback and being able to be criticized	Dyess et al. (2015), Buell (2014), Wicks and Buck (2013)
coping mechanisms (breathing techniques; balance between professional and private life; setting of priorities)	Bright (1997), Cline (2015), Kim and Windsor (2015), Dyess et al. (2015)
*External* factors of healthcare leaders’ resilience	
positive relationships with family and friends	Bernard (2019), Kim and Windsor (2015)
networks	Cline (2015), Hudgins (2016)
role models	Wicks and Buck (2013)
mentor or coach	Kelly et al. (2016), Grant et al. (2009), Westcott (2016)
time for yourself (e.g. to reflect) and time to relax	Bernard (2019), OʼConnor and Batcheller (2015), Wicks and Buck (2013)
good night’s sleep (especially for nurses on shifts)	Bernard (2019)
experiences in different areas, including work experience	Cline (2015)
experience in coping with crises	Hamilton (2015), OʼConnor and Batcheller (2015)
*Organizational* factors of healthcare leaders’ resilience	
organizational culture (mutual respect, equality and role clarity)	Kelly et al. (2016)
stability and security	Kim and Windsor (2015), OʼConnor and Batcheller (2015)
a close relationship between superiors and employees	Kelly et al. (2016)

Although various factors have been identified in previous research as promoting healthcare leaders’ resilience, to our knowledge, no comprehensive classification or systematization of these influencing factors, for instance, in terms of a systematic framework, has been forthcoming. Considering the importance of this topic, we urgently need to know how resilience in these systems can be promoted. Examining resilience within healthcare systems should most appropriately start by investigating healthcare leaders’ resilience because their resilience is not only affecting the leaders themselves but also, through a trickle-down effect, their employees and the entire organization (e.g., Avey et al. 2011; Foerster and Duchek 2017; Gooty et al. 2009; Walumbwa et al. 2010). At the individual level, leaders’ resilience promotes well-being (Grant et al. 2009), and joy and satisfaction at work (Bernard 2019; Qing et al. 2020). It also reduces any intention to change jobs (Hudgins 2016), as well as relieving depression, anxiety, and stress (Grant et al. 2009). Furthermore, if leaders are resilient themselves, they are more likely to act as role models and to promote the resilience of their teams, which again contributes to higher resilience and better performance across the entire organization (e.g., Cline 2015; Hillmann 2021). Looking at it the other way around, if the leader is unable to cope effectively with the crisis, it is unlikely that the team can do so, probably leading to an increase in sick days and staff departures (Buell 2014; Wicks and Buck 2013). Thus, leaders need to develop both their own resilience as well as that of those around them (Bernard 2019).

## Method

### Research aim, sample, and procedure

Based on resilience research in the leadership context (e.g., Foerster and Duchek 2017, 2018) and in the work context (e.g., Cooper et al. 2013; Kossek and Perrigino 2016), our empirical study aimed to shed light on the challenges healthcare leaders perceive and context-specific factors influencing healthcare leaders’ resilience.

In sum, we conducted interviews with 20 professionals, of whom 35% (7) were physicians, 55% (11) were nurses, and 10% (2) were nonclinical professionals, for instance, HR managers (see Table 2).

**Table 2:** Interview participants

Interview	Work area	Work place	Leadership level
1	Medical i.e. nursing	Hospital	Middle
2	Medical i.e. nursing	Hospital	Upper
3	Medical i.e. physician	Hospital	Upper
4	Administrative/non-clinical	Hospital	Upper
5	Medical i.e. physician	Hospital	Upper
6	Administrative/ non-clinical	Assisted living	Upper
7	Medical i.e. physician	Hospital	Middle
8	Medical i.e. nursing	Hospital	Middle
9	Medical i.e. physician	Hospital	Upper
10	Medical i.e. nursing	Welfare organization	Lower
11	Medical i.e. nursing	Hospital	Upper
12	Medical i.e. physician	University hospital	Middle
13	Medical i.e. nursing	Hospital	Upper
14	Medical i.e. nursing	Hospital	Lower
15	Medical i.e. nursing	Hospital	Middle
16	Medical i.e. nursing	Hospital	Middle
17	Medical i.e. nursing	Hospital	Middle
18	Medical i.e. physician	University hospital	Upper
19	Medical i.e. physician	University hospital	Middle
20	Administrative/non-clinical	University hospital	Upper

Data collection took place between March and May 2019. Regarding the sample, 50% (10) of our participants were working in upper leadership positions, while 40% (8) worked in middle and 10% (2) in lower leadership positions. Eighteen of these healthcare leaders were employed in hospitals situated in Germany, one in a welfare organization, and another one in an assisted living facility. Of the sample, 60% were female, and the participants averaged 50 years of age (36–63 years). Our healthcare leaders were responsible for an average number of 145 employees, ranging from 9 to 800 employees. With the exception of one participant, all the leaders had worked in the healthcare sector throughout their careers, while rarely changing their employer. On average, our leaders had worked for fewer than three different institutions, and 25% (5) had worked at one organization for their entire professional life.

To examine our research questions, we chose an open and narrative approach that also allowed for the exploration of new knowledge. The interviewees were storytellers rather than respondents, and the agenda was open to change depending on the interviewee’s experiences (Hollway and Jefferson 2008). We used a semi-structured interview guideline addressing particular healthcare leaders’ challenges and context-specific resilience factors, whereby we explicitly asked about individual factors, situational factors, and behavioral factors. The guideline helped us to address all relevant themes and ensured the comparability of the conducted interviews. However, the guideline was not used as a deterministic schedule. The focus of the conversation and the order of questions were flexibly handled. To gain profound and comprehensive insights, the participants were encouraged to use concrete examples of critical situations to illustrate their answers (‘critical incident technique’; Flanagan 1954). In sum, we gathered a total of 11 h of interview material with the shortest interview lasting approximately 21 min and the longest 56 min (35 min on average). The interviews were voice-recorded and transcribed afterward. To ensure our participants’ confidentiality, we replaced all names with numbers (01 to 20) and removed any other identifying information.

### Data analysis

Our data analysis draws on the qualitative content analysis approach (Miles and Huberman 1994) and involves both inductive and deductive elements. Since research on both health care leaders’ and leaders’ resilience in general is limited (Foerster and Duchek 2018) but the context-dependency of the resilience construct is high (e.g., Fletcher and Sarkar 2013; Kossek and Perrigino 2016), we decided to rely for our research on one of the few approaches that systematically try to understand the influencing factors of leaders’ resilience, namely the framework of leaders’ resilience by Foerster and Duchek (2017). Based on Cooper et al.’s (2013) workplace resilience framework, the authors deductively derived three main categories of leaders’ resilience factors: individual, situational, and behavioral. In more detail, *individual factors* within the original study of Foerster and Duchek (2017) consist of both traits and abilities. “Traits are comparable and largely stable features of an individual, representing the person’s fundamental personality” (Foerster and Duchek 2017: 11) such as self-confidence or optimism. In contrast, “abilities have been acquired by the participants and were not present from the beginning” (Foerster and Duchek 2017: 11), for example, communication skills. Further, *situational factors* can be summarized as “external support and resources” (Foerster and Duchek 2017: 6; Cooper et al. 2013) and include private factors such as family and friends as well as work-related factors such as a positive work climate. In addition, *behavioral factors* are those resilience factors that underlay the resilience process, and thus result from the application and expansion of “existing resilience resources” (Foerster and Duchek 2017: 6; Cooper et al. 2013). Examples are a reflective acting at the personal level or an open communication within the team. Referring to our study of health care leaders’ resilience, the factors within the main categories were derived inductively from the data material.

The data analysis can be roughly divided into the following stages. In the first stage, we analyzed our data following the idea of grounded theory (Strauss and Corbin 1998) and the Gioia methodology (e.g., Gioia et al. 2012). We identified factors that make resilience necessary in terms of the challenges the healthcare leaders perceived. We then identified resilience factors that helped the leaders to overcome critical situations. We compressed the empirical data by assigning relevant text segments (sentences or paragraphs) to these factors (coding). In the second stage, we analyzed the findings in detail. As a result, within each main category (e.g., situational factors), we found various subcategories (e.g., network and exchange). The definition of these categories and the assignment of factors to categories were refined in several feedback loops until the final classification system was established. In the third stage, we focused on the interrelationships among the identified factors and categories. The data analysis was performed with MAXQDA. Table 3 provides an overview of the categories of healthcare leaders’ challenges, their resilience factors and exemplary quotes.

**Table 3:** Categories of healthcare leaders’ challenges and resilience factors

	Exemplary quotes
Type of challenge	
Challenges	
Work-related challenges	“So it’s extremely changeable, no two days are the same. There is virtually no routine, which in turn is extremely demanding.” (19) “If, despite all efforts and sometimes very good results of the surgical procedure, patients die, that is of course always bitter (…) you also bond with the people you deal with.” (7)
Leadership challenges	“These are simply challenging situations in leadership. So 450 employees are not always healthy, motivated, optimistic, in a positive mood, but there are always some who are really annoyed that they have to be at work and that they have to work here. And they are maybe, well not maybe but they are sometimes of course also annoyed by a higher cycle frequency or a higher performance that you have to bring.” (2)
Type of resilience factor	
Individual factors	
Positive attitude	“I think being a pessimist while holding a leadership position won’t work. This fundamental belief that something is possible, achievable and can be solved, with [this attitude] I always approach all things. It’s really terrible, if you think (…) we won’t make it. [With this attitude] you can’t motivate anyone else to do anything.” (2)
Confidence and self-efficacy	“I really perceive myself as efficient, also as self-efficient. You can support patients in crises, in certain phases of life that are not so pleasant, you can accompany them well.” (17)
Personal skills and abilities	“The [leaders] must (…) have this will to power themselves and say that I want the area to be worked as I imagine it to be.” (6)
Social skills	“I’m not a lone wolf, I don’t have to do it all by myself, so I get help from the team. (…) Then it becomes easier, some problems are easier on many shoulders.” (14)
Openness to learn and change	“Openness to change and new approaches to solutions, to other ways, that is, I believe, essential if you cannot change the framework, that you can change the way in which you provide services and control processes, I believe that is important.” (2)
Situational factors	
Work content	“I think that has to do with the fact that if you enjoy doing something, it doesn’t feel like a burden.” (1)
Atmosphere and support at work	“I also have two or three friends among my colleagues here and that’s simply a high resilience factor in work.” (19)
Social support in private life	“Well, this is a world of its own that is so rich that it strengthens you. I don’t have to discuss with my friends what I’m experiencing [at work]. (…) It’s more like that (…) you do things, we go to exhibitions with friends, we go on cultural trips. It’s just so rich and so invigorating that it works by itself.” (5)
Network and exchange	“Networking is very important; to network on the same level, to know colleagues on the same hierarchy level and to be able to exchange ideas with them in a trusting manner; to be able to say openly when something is really annoying and to recognize that the other party feels exactly the same way. That alone is really helpful (…) And then to exchange possible solutions (…) networking is something very important.” (2)
Flexibility	“I have a budget and I can use this budget freely (…) That of course gives you great freedom in making decisions, knowing that I can decide that, nobody questions that.” (2)
Feedback and recognition	“It helps a lot when you have successes, when you get that back, when you get praised. That confirms that what you are doing is right. The more you have this, the more confident you become, and the less problems can knock you over.” (16)
Health	“(…) the whole thing wouldn’t work (…) if you are not healthy in body and soul (…) to be able to do something like this.” (9)
Behavioral factors	
Accepting the situation	“Generally there are always unforeseen situations (…). Then of course, you have to look at, ‘Oh, how do you get that organized now.’ In general, nothing knocks me off my stool so quickly.” (01)
Analytical procedure	“I can also prioritize what is important and what is not important, I can classify it according to urgency. I think I have organized myself well in terms of time.” (15)
Differentiation	“And if you have the basic attitude that the world keeps turning, no matter what happens, that it is relatively irrelevant to the bigger picture, then it can sometimes be easier to work.” (4)
Compensation	“I think I can switch off well. So then, I also know what will help me. If it is really bad, just get out, run, walk until your head is clear again. Or culture, movement in the broadest sense, sometimes dancing or something, so I know what is good for me when I have to reduce stress.” (9)
Open communication and interaction	“We are relatively open, I think, here in the team on how we treat each other, we also talk about things so that things don't go wrong. I am a fan of direct communication, I actually address that too.” (14)
Reflection and change behaviors	“and then to reflect, why am I reacting like that […]. I find that very helpful.” (17)

## Findings

To better explain our healthcare leaders’ challenges and context-specific resilience factors, we will start the findings section with a short explanation of the healthcare leaders’ main tasks at work. The participants described three different types of tasks. First, *administrative tasks* often include, among other things, organization, planning, concept development, and billing. Second, *leadership tasks* range from the acquisition of employees and drafting of the duty roster to direct employee management and the responsibility for their further training. Third, *nursing and medical tasks* are mainly related to patient care but also include research and scientific work. While all respondents were entrusted with the first two tasks, the nursing and medical tasks were described as gradually being reduced as the leadership tasks increased. About half of our participants stated that they no longer worked with patients or that such a responsibility was not part of their job description. Although these respondents all worked in nursing or management, the physicians also declared that their clinical activity had been decreasing as the managerial responsibility increased.

### Challenges

Our participants reported several challenges that they had to conquer, sometimes on a regular basis, and which they perceived as stressful. Apart from widespread challenges (e.g., globalization, digitalization) and individual challenges (e.g., family problems), which occur irrespective of the context, our participants also stressed several occupation-specific stressors arising from their work as healthcare leaders, namely challenges arising from work as a healthcare professional and challenges arising from their work as a leader.

#### Work-related challenges (administrative, nursing/medical)

Healthcare leaders’ challenges arise from their responsibilities that can be divided into three main areas: (1) nursing and medical, (2) administrative, and (3) leadership. Although some leaders’ challenges are independent of the occupation, such as cost pressures, lack of time, or staff shortages, others are specific challenges arising from working in the healthcare context. Working in healthcare means working with different people from different groups every day, including patients, relatives, colleagues, and superiors. Some of our respondents stated that their daily routine is hardly plannable since illness among employees, emergencies, or unpredictable situations with patients, and different organizational or administrative tasks ensure that the working day is often fragmented and unpredictable. Related to the work with people, other health care-specific challenges are the experience of heavy fate of patients due to serious illnesses or accidents, risk situations, and complications, which result from working with patients. About half of our healthcare leaders named difficult patients and the experience of difficult fates as challenges. Patients often have special requirements and express their dissatisfaction clearly. Although patient complaints and dealing with relatives are part of everyday life at work in the healthcare system, they repeatedly lead to stressful situations, whereby the health status of individual patients—for instance, those suffering from dementia or obesity—can make dealing with patients more difficult. Another aspect that in particular leads to mental burdens results from dealing with death, special life situations, or traumas experienced by others. Some of the healthcare leaders reported cases that deeply touched them and whose imprint was still fresh in their minds. Participants also mentioned risk situations and complications when treating patients as everyday work challenges. Particularly when the patient’s life is at stake, the patient is harmed, or errors have to be dealt with, our interviewees reported to be highly stressed.

An extensive workload, information overload, and ever-changing and fast-moving circumstances were challenging our healthcare leaders on a daily basis. The constant change and the fast pace in communication were leading to new influences and demands, which a third of our leaders experience as stressful. More than half of them mentioned a high workload with several causes, including various tasks that have to be completed and deadlines that have to be met. All these demands can consume a considerable amount of time, which in turn often requires working overtime. In addition to cost pressures as a crucial healthcare leaders’ challenge, a steadily increasing workload has also been caused by demographic change, with older and morbid patients and a skilled labor shortage, as our participants stated. Our interviewees stressed that bureaucracy requires extensive documentation and the adjustments to the practical work are enormous in the healthcare system. They named compliance with and implementation of legal requirements as a challenging factor. These requirements decrease the scope of the leader’s influence so that problems often cannot be solved, which in turn leads to a feeling of being at the mercy of someone else.

#### Stress due to the leadership challenges

Working in a leadership position is also a challenge for the interviewees. Most of them indicated that stress accompanied personal responsibility. They reported that he/she often must act as a mediator when conflicts arise between team members. For two-thirds of our healthcare leaders, repeated interpersonal conflicts cause stress. They also mentioned conflicts with colleagues operating at the same hierarchical level or those who see themselves as better off, whereby such interpersonal conflicts result in an increased stress level for the leader regardless of its trigger. As a major problem, if not the main one, our healthcare leaders described staff absenteeism and a general staff shortage. While almost all our participants described the same problem, the staff shortage affects them in various ways. First, they explained that it is difficult to provide adequate coverage by replacing a person who is ill or is not allowed to work due to, for instance, pregnancy. In these cases, other employees have to step in or be assigned additional duties to ensure safe patient care. Our healthcare leaders reported that they have to avoid beds that are not usable (due to staff shortage) because this would cause economic losses. If no other option is available, our leaders themselves have to fill the empty position and, are thus unable to perform their normal duties adequately. These situations, especially when no substitute can be found and staff shortage becomes chronic, cause high stress for our healthcare leaders, keeping in mind that a chronic staff shortage might be life-threatening in the healthcare sector. In addition, our participants reported that structural changes in recent years have increased the number of employees and locations for which a manager is responsible, resulting in additional workload. Furthermore, the chronic staff shortage creates additional challenges in terms of acquisition and retention. To attract and retain employees, the healthcare leaders reported that they have to be attractive as an employer, make offers, and have a good reputation. However, this position is difficult to maintain if overwork leads to dissatisfaction. Our respondents also reported that well-qualified employees have numerous employment possibilities and thus make higher demands of prospective employers. This leads to fewer applications per position, and the situation that sometimes less qualified people have to be hired. Three quarters of our healthcare leaders describe their task of motivating employees, especially when they are frustrated due to staff shortages and overtime, as stressful. They also reported that they are often faced with employees belonging mainly to Generation Y (1980–1994) or even Z (1995 onwards), who are shaped by different values, ideas, and work concepts. According to one-third of our participants, such differences lead to tensions within the team. Based on their own experience and professional careers, healthcare leaders cannot understand this ‘new’ work attitude. Nonetheless, they have to find access to these employees, which poses a challenge for them.

In addition, most of our interviewees felt pressured from being caught in a triangle between the economic goals of the organization (i.e., performance, political targets), their employees (i.e., employee satisfaction), and their patients (i.e., quality of patient care), whereby the interests of these groups are, at least to some extent, mutually exclusive. Resulting from this dilemma, the leaders sometimes have to represent issues that they do not actually support, always balancing the interests of the various stakeholder groups, always operating at the interface. Apart from the interests of various stakeholders, healthcare leaders have to fulfill different roles during their working day, which can lead to intrapersonal conflicts, thus acting as yet another stressor for the healthcare leaders.

### Context-specific factors influencing healthcare leaders’ resilience

The resilience of healthcare leaders is influenced by various factors, which can be divided into individual, situational, and behavioral factors. In the following section, we explain the main factors within each of these categories.

#### Individual resilience factors of healthcare leaders

This category includes (1) a positive attitude, (2) confidence and self-efficacy, (3) personal skills and abilities, (4) social skills, and (5) openness to learn and change.

##### Positive attitude

Most of our healthcare leaders see their positive attitude as a foundation for their resilience. They explained that they assume that all tasks and problems can be solved somehow, and they always try to see the positive side in people as well as the challenges and retain a certain degree of humor. According to our participants, this fundamental positive attitude not only helps them to navigate through critical situations but also trickles down to their employees.

##### Confidence and self-efficacy

Even though a positive attitude seems to be fundamental for the healthcare leaders’ resilience, being able to see things positively also depends, at least to a certain degree, on the individual’s self-confidence. According to our healthcare leaders, being self-confident is a condition for asserting yourself in a leadership position and, when needed, against others. It also includes trusting one’s own abilities, which in turn leads to the conviction that any challenge can be mastered.

##### Personal skills and abilities

Our healthcare leaders named a certain determination or goal-orientation and the ability to assert oneself as important to their resilience. Determination implies perseverance, which means that the leaders do not give up and are able to endure even unpleasant situations. After all, they have already reached a leadership position through the exercise of their determination. The ability to assert oneself is needed, in turn, to enforce decisions and also to distance oneself, which was also mentioned as an important resilience factor. Furthermore, professional skills offer the opportunity to develop various personal resources that in turn positively influence resilience. Our healthcare leaders described certain conceptual skills, such as planning, structuring, organizing, coordinating, and anticipating, as being positively related to their resilience. They also mentioned problem-solving skills as an important resilience factor.

##### Social skills

Our healthcare leaders further explained that social skills, especially empathy and communication skills, are highly important components of their resilience. The leaders argued that by perceiving other people’s emotions, they are able to understand, for instance, people’s reactions or claims. Concerning effective communication, leaders especially need negotiation skills but also have to convey information in the right way, especially when conducting employee performance reviews or when delivering diagnoses to patients. In essence, effective communication can help to resolve conflicts and to strengthen multi-professional teams, using a certain degree of politeness, friendliness, and transparency that always has to be maintained. Closely related to these skills are team skills. Our healthcare leaders underscored the importance of being able to delegate tasks while supporting and shaping teamwork. As they explained in the interviews, it is important to involve their employees and to enable them to deal with challenges. The healthcare leaders also made clear that they had to place a certain amount of trust in their employees. Finally, the willingness to seek and accept help has been underscored as an important aspect enabling teamwork and reinforcing their own resilience. Getting assistance can enable managers to survive situations in which they did not know what to do. The fundamental attitude that their work is not a one-man show helped to fortify the resilience of the healthcare leaders.

##### Openness to learn and change

Being open to others and their ideas and suggestions was described as important to the participants’ resilience and as a prerequisite to their own willingness to learn because learning referred to numerous aspects of their work. In more detail, the healthcare leaders underlined the importance of staying up to date in their area of expertise, to learn from criticism and experience. Closely related to the participants’ willingness to learn is also their willingness to change, which was named as an important resilience factor by our healthcare leaders. Only if the leaders are able to question the status-quo as well as their own actions are they able to encounter new possibilities, take advantage of them, find ways out of difficult situations, and implement what has been learned.

#### Situational resilience factors of healthcare leaders

This category includes (1) importance of work, (2) atmosphere and support at work, (3) social support in private life, (4) network and exchange, (5) flexibility, (6) feedback and recognition, and (7) healthy lifestyle.

##### Importance of work

The interviewees stated that their work is important and that they find meaning and joy in performing it. They believe that they can make a difference and that supporting others is the way to do it. They are doing their jobs with passion and feel at home in their jobs. They show a high degree of intrinsic motivation. With this attitude, they can better handle the challenges of their job.

##### Atmosphere and support at work

An atmosphere characterized by good relationships, including mutual care, loyalty, and respectful communication, is perceived as supportive and strengthening. In addition to the general work climate, our leaders described specific reference persons and good individual relationships within the organization as strengthening their resilience. Positive relationships ensure that one feels comfortable and enjoys work. They are part of an intra-organizational support system and provide social support.

##### Social support in private life

Beyond that, family and friends were also considered as important resilience factors. According to our respondents, their private environments provided stability, exchange, advice, and support. Friends and family were often mentioned in relation to activities that enable our healthcare leaders to balance, switch off, or change their perspectives. In this sense, our participants enjoyed getting together with families and welcomed the input offered by them on various topics and experiences. To have a strengthening effect, the respondents stressed, the stability of these relationships and the condition that they do not add any additional problems are essential components.

##### Network and exchange

A sustainable network is an important resilience factor. A network can be developed with colleagues across professional groups as well as with internal and external contacts, for instance, by treating a patient together or with former colleagues. Considering that leaders are often in lonely positions, our healthcare leaders in particular stressed the benefit of relationships with people at the same hierarchical level or in the same position. Exchanging with people in the same position helps the healthcare leaders realize that others have the same problems as themselves. In addition, they gain from discussing ideas and solutions with their peers, especially in precarious situations.

##### Flexibility

Our healthcare leaders explained the desirability of flexibility, first, in terms of working time, space, and situation, but also referring to a certain degree of personal freedom. A degree of flexibility positively influences their resilience because it helps them to meet different demands and challenges. Leaders who are free to organize their appointments and who have the option to work from home or with flextime are, according to our participants, better able to combine private and professional issues. Although hidden dangers can emerge from flexible working hours (e.g., lengthy stints at the office), our healthcare leaders predominately stressed the positive aspects of flexibility. They argued that having the freedom and responsibility for their professional decisions leads to greater satisfaction and creates new options for solving problems.

##### Feedback and recognition

Although patient feedback is often given very directly and immediately when healthcare professionals are working in a ward, patient contacts decrease for those entering leadership positions and so does the feedback. Nonetheless, receiving feedback has remained important for our leaders, and might come, for instance, through their teams or directly from patients in the case of leaders operating in middle or lower leadership positions. Yet leaders do not just need support through feedback. Support, in particular from upper hierarchical levels, can come through the quick handling of tasks, as organizational support, or receiving an open ear for problems. As our healthcare leaders highlighted, they need the organization to support them, not to monitor them.

##### Healthy lifestyle

Finally, our healthcare leaders mentioned their own health as an important resilience factor. Health includes both one’s physical health and also mental health. Sufficient restful sleep, a healthy lifestyle, and a certain inner balance are essential to maintaining one’s well-being. Overall, satisfaction, happiness, and balance contribute to stability, which in turn is important so that critical situations do not throw one off course easily.

#### Behavioral resilience factors of healthcare leaders

Within this category, we can see how the individual and situational resilience factors come together, interact, and find expression in different behaviors that help our leaders to cope effectively with different challenges and crises in their professional life. The category includes the following behaviors: (1) accepting the situation, (2) analytical and structured procedure, (3) differentiation, (4) compensation, (5) open communication and interaction, and (6) reflection and change behavior.

##### Accepting the situation

Our interviewees stated self-confidence and a positive attitude assists in maintaining their serenity and accepting whatever situation they are facing. Acceptance of the status quo helps them to stay calm and rational amid crises, when any sign of restlessness on their part could trickle down to the employees.

##### Analytical and structured procedure

Some healthcare leaders stated that having a routine and action strategies in their professional work gave them security and helped them deal with critical situations. According to our interviewees, analyzing a problem, developing a solution and using available resources adequately had developed into a proven strategy for them.

##### Differentiation

Our healthcare leaders stated that it is important for them to maintain some distance between critical situations and themselves. They need to distance themselves from tasks, demands, or the work itself, as well as from failures and stressful situations, but also from emotions and hasty reactions. Keeping a distance enables them to achieve a certain degree of objectivity and rationality that helps to cope with situations. In line with this differentiation strategy, our healthcare leaders explained that they strengthen their resilience by being relativistic. Looking at the big picture assures our leaders that not everything depends on them, and a problem is just a problem. They also realize that their job is just a job and that life has to offer much more.

##### Compensation

Closely related to differentiation is the topic of compensation. Our healthcare leaders explained that they try not to do any work at home and “flip the switch” after work and concentrate on the family by shelving any tasks and their phone. Many of the interviewees say they consciously create time for themselves, taking breaks to gather new energy or to escape stressful situations, even if only for a short time. Any activities they perform are for their own benefit and according to their individual preferences, such as sleeping, taking a vacation, or rewarding themselves in some way. Such activities were felt to contribute to their regeneration and strengthening. In their private lives, the healthcare leaders referred to hobbies or activities that specifically enabled them to switch off and regain their balance. Thus, each had a way of clearing the mind, for instance, through sports, culture, meeting friends, or enjoying nature. Just as individual as the activity itself is the regularity with which it is carried out. Despite these differences, it seemed to be important for all the healthcare leaders to spend their free time meaningfully so as to reduce their stress. Furthermore, they emphasized that dealing with completely different topics and doing completely different things than in their profession was particularly helpful in boosting their resilience.

##### Open communication and interaction

Another behavioral resilience factor that the healthcare leaders saw as helpful was open interaction with their team members. They explained that being open in terms of threats, emotions, one’s own fallibility, and changes involving the team led to a higher degree of acceptance and thus provided support for themselves. In relation to the many conflicts described above, our leaders also considered facing and mitigating those conflicts to be as important as suggesting and making compromises. For this purpose, open communication was essential. However, our participants also said that they only intervened when the conflicts were brought to them for resolution.

##### Reflection and change behavior

Our healthcare leaders stated that reflection, in terms of clarifying things for themselves and understanding new situations, helped them to navigate through critical and unusual situations. Even though our healthcare leaders relatively seldom addressed this aspect, it seemed to be a definite means of increasing resilience. It allows leaders to learn from experienced situations, to adapt, and to become stronger than before. However, it can be assumed that leaders often reflect unconsciously and therefore do not describe this behavior explicitly as a resilience factor. In addition, our participants also stressed that through a change of their perspective, for instance, by perceiving and valuing the opinion of others, they could get new directions when solving a problem and thus achieve positive learning effects.

## Discussion

This study expands what we know about resilience of healthcare leaders, a research field that has been widely neglected so far. This is quite surprising since we know that especially in managing uncertainty, organizational crises, and stressful situations, the role of the leader has been mentioned repeatedly (e.g., Pearson and Clair 1998). We argue that leaders need resilience since healthcare teams depend heavily on their leaders (e.g., Dewey et al. 2020). If leaders are resilient themselves, they are more likely to act as a role model and to promote the resilience of their teams, which again contributes to a higher resilience and better performance of the entire organization (e.g., Cline 2015; Hillmann 2021).

By iterating our empirical data and the relevant literature (e.g., Cooper et al. 2013; Foerster and Duchek 2017; Kossek and Perrigino 2016), we were able to develop an *integrative framework of healthcare leaders’ resilience*. Embedded in the healthcare organization context, healthcare leaders’ resilience as an outcome stems from the interaction of individual and situational factors leading to resilient behavior (resilience as a process; Cooper et al. 2013; Kossek and Perrigino 2016; Foerster and Duchek 2017).

**Fig. 1:**
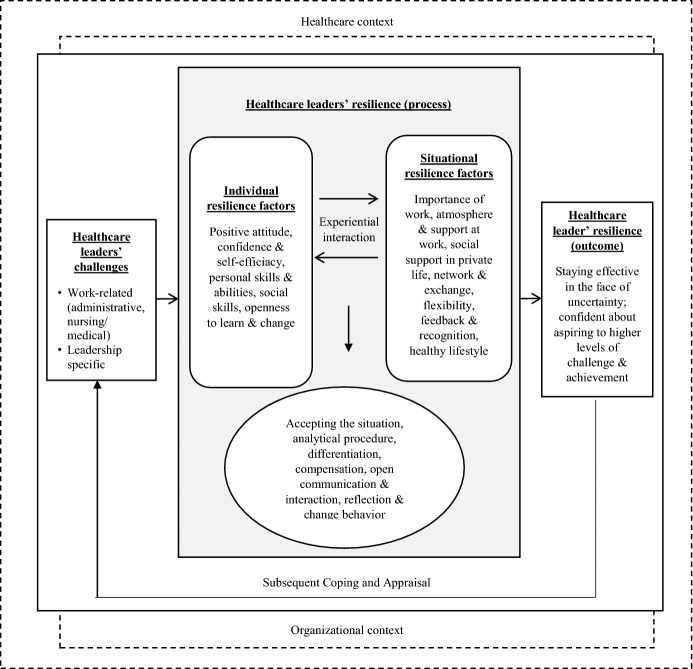
An Integrated Framework of Healthcare Leaders’ Resilience (further development based on Cooper et al. 2013; Foerster and Duchek 2017; Kossek and Perrigino 2016)

Inspired by previous research, we organized the specific resilience factors of healthcare leaders into three general categories: *individual, situational,* and *behavioral factors*. We systematized these factors into an integrative framework (Fig. 1).

Some of our identified factors were also found in earlier studies (see Table 1), although none of them has been identified as part of a broader framework.

Apart from the single resilience factors within the three general categories (individual, situational, and behavioral factors), we have also found that individual and situational healthcare leaders’ resilience factors interact on an experiential basis. *Experience*, as an interplay of individual and situational factors, strengthens resilience over one’s life span. Childhood experiences, crises, and strokes of fate are described as strengthening individual resilience. Our healthcare leaders frame professional setbacks, critical situations, and certain events positively to find a new way to deal with their lives. Positive experiences and success have also been useful in strengthening their resilience. Many of our respondents stated that past professional and life experiences helped them to cope with specific stressors. This is also confirmed in other studies. According to Kelly et al. (2016), leaders can develop resilience through their individual experience of challenging situations. Success experiences in particular can lead to an increase in self-confidence and thus increase resilience (Kim and Windsor 2015), but even experiencing crises such as losing one’s job cannot take away the experience gained, and overcoming the crisis can create additional resources (Hamilton 2015; OʼConnor and Batcheller 2015; Hillmann 2021).

Apart from this positive connotation, we also identified a potential threat in the sense of a *vicious circle of stress* that deeply endangers healthcare leaders’ resilience and results from the hardly solvable triangular relationship among the three duty areas mentioned above. Different challenges can influence each other and, in the worst case, reinforce each other. For instance, aging and multimorbid patients increase the need for nursing staff because patients require more time and sometimes also more personnel. If the workload increases and no extra staff is hired, employees have to take on more work, which in turn engenders frustration and dissatisfaction. Disappointed employees are more likely to call in sick, which further exacerbates the problem of staff shortages and makes it more difficult for the leaders to motivate and retain these employees. Since the acquisition of new employees also depends on the reputation of the institution, general and obvious employee dissatisfaction decreases the attractiveness of having the institution as an employer, leading to more vacancies that cannot be filled and, thus, to heavier workloads and frustration for the remaining employees and leaders. What happens is a self-reinforcing negative cycle containing several of the above-mentioned challenges of healthcare leaders. In addition to the internal factors fueling this cycle, external factors can also worsen the situation. For instance, experiencing information overload, keeping a fast pace with 24-h availability and legal changes slowly but surely lead to an ever-increasing workload. This might be an explanation why even health care professionals with high resilience levels exhibit burnout symptoms (West et al. 2020). Thus, even if resilience might be associated with lower burnout levels (West et al. 2020), it does not free politics and organizations to reduce institutional and organizational constraints that might cause burnout and other stress-related diseases, a development that has been particular highlighted in the COVID-19 pandemic (e.g., Hlubocky et al. 2021; Madara et al. 2021; Ripp and Shanafelt 2020; Shanafelt et al. 2019; Sikka et al. 2015).

Like any research, our study has its limitations. We used a qualitative study and interviewed 20 healthcare leaders in Germany. As a result, our findings provide insights into the complex topic of healthcare leaders’ resilience and relevant resilience factors, which we used to develop our integrated framework. Although these findings are shaped by a country-specific context and cannot be generalized, they build the basis for more comprehensive future research in this field. We already know from psychological studies, especially with young people (e.g., Fletcher and Sarkar 2013; Ungar 2008), that resilience is a complex construct and more importantly, that it is a culturally and contextually embedded construct. Therefore, it would be interesting to explore the influence of different structural conditions of the health care sector in different countries on the resilience of healthcare leaders. It would be interesting to learn what common patterns and differences exist. Thus, future studies should be conducted in different national contexts and in particular focus on the individual prerequisites of resilience in connection with organizational constraints, and structural factors and framework conditions (e.g., Shanafelt et al. 2019).

Furthermore, future research could differentiate among three professional groups, i.e., physicians, nurses, and nonclinical leaders, to provide more specific insights into relevant context-dependent resilience factors. Based on our findings, we also assume that causal relationships exist between the identified resilience factors, but we cannot confirm the validity of this notion by means of this qualitative study. For this purpose, standardized quantitative research with a larger number of participants would be needed.

## Practice implications

Speaking with healthcare leaders during the current COVID-19 crisis underlined that being resilient is a prerequisite for overcoming this crisis in an effective and healthy manner. Due to the intensity of the current crisis, healthcare leaders often reported that they were drawing from resilience resources they had built up in the past. The crisis also illustrates the impact of healthcare leaders’ resilience on their organization. In this sense, resilient healthcare leaders who relied on their network connections to China and Italy may have better prepared their organizations for the looming crisis. Therefore, healthcare leaders need to strengthen their resilience to better cope not only with minor everyday crises but also with more substantial ones. Based on our integrated framework of healthcare leaders’ resilience, the leaders’ behaviors provide a good starting point for an effective healthcare leaders’ training program. Although plenty of possibilities to promote leaders’ resilience exist, active learning methods, such as cooperative learning, role-plays, or simulations (e.g., Snyder 2003), are most appropriate for training people in resilience behaviors. Nonetheless, which training methods are best suited must be carefully evaluated beforehand, especially depending on the healthcare leaders’ situation and training goals. However, it should be recognized that even if such a program increases the healthcare leaders’ ability to handle stress and to remain healthy even in stressful situations, resilience is not an ultimate remedy, and certainly has its limits. Thus, healthcare institutions must determine the extent to which existing challenges can be reduced and how, particularly in the face of the above-described vicious circle of stress, the three healthcare leaders’ task areas (i.e., medical/nursing, administrative, and leadership) can be harmonized. Although most of the problems (for instance, a lack of qualified medical stuff) are structural problems and systemic challenges, which were known long before the crisis, the COVID-19 crisis functioned as a ‘magnifying glass’ (e.g., Foerster and Fuereder 2021; Madara et al. 2021). One interesting possibility may be the implementation of health care chief wellness officers (CWO), who “focus primarily on improving their organizations’ work environment and culture … to address what is wrong with the practice environment, not to make individuals better able to tolerate a broken system.” (Ripp and Shanafelt 2020: 1354).

## Data Availability

Not applicable.
